# Systemic vascular leakage induced in mice by Russell’s viper venom from Pakistan

**DOI:** 10.1038/s41598-018-34363-1

**Published:** 2018-10-31

**Authors:** Alexandra Rucavado, Teresa Escalante, Erika Camacho, José María Gutiérrez, Jay W. Fox

**Affiliations:** 1 Instituto Clodomiro Picado, Facultad de Microbiología, Universidad de Costa Rica, San José, Costa Rica; 2 School of Medicine, University of Virginia, Charlottesville, VA 22959 USA

## Abstract

Envenomings by some populations of the Russell’s viper (*Daboia russelii*) are characterized by a systemic capillary leak syndrome (CLS) which causes hemoconcentration, and is associated with the severity of envenoming. We adapted a model of CLS in mice by assessing hemoconcentration. The venom of *D. russelii* from Pakistan, but not that of another viperid, *Bothrops asper*, induced hemoconcentration and an increment in vascular permeability, being devoid of hemorrhagic activity at the doses tested. These findings reveal a dichotomous pattern of vasculotoxicity in viperid snake venoms. This difference might depend on variations in venom composition, especially regarding metalloproteinases (SVMPs), which are low in Pakistani *D. russelii* and high in *B. asper*. Inhibition of SVMPs and phospholipases A_2_ in *D. russelii* venom did not abrogate hemoconcentration. An hemoconcentration-inducing fraction was obtained by chromatography, which contains vascular endothelial growth factor (VEGF), a known potent inducer of increment in vascular permeability. Exudates collected from tissue injected with venom also induced hemoconcentration, and the effect was inhibited by antivenom. However, the amount of venom in exudate required to induce the effect is low, as compared with venom dissolved in saline solution, hence suggesting that endogenous proteins present in the exudate, probably inflammatory mediators, potentiate the effect.

## Introduction

Envenomings by snakes of the family Viperidae (true vipers and pit vipers) are generally characterized by vascular toxicity leading to local and, in severe cases, systemic hemorrhage^1,2^. Hemorrhage is primarily a consequence of the action of zinc-dependent metalloproteinases (SVMPs) in the microvasculature^3,4^. Bleeding is potentiated by blood incoagulability and platelet alterations, generated by the action of procoagulant enzymes, which induce a consumptive coagulopathy, and by proteins that inhibit platelet aggregation and/or cause thrombocytopenia^1,2,5^. Further, local and systemic bleeding may lead to hypovolemia and cardiovascular shock^1,2^. An outcome associated with profuse bleeding is generally a drop in hematocrit and in hemoglobin concentration^6^. Hemodynamic disturbances are also induced by increments in vascular permeability, mediated by the direct action of venom components and also by endogenous mediators released as a consequence of tissue response to damage.

In contrast to this general pattern of vascular toxicity in viperid snakebites, envenomings by some populations of Russell’s viper, *Daboia russelii*, a species of wide distribution in south Asia, are characterized by a unique clinical manifestation, i.e. systemic capillary leak syndrome (CLS)^7^, which causes hemoconcentration and hemodynamic alterations and has been associated with the severity of envenoming and high mortality^8,9^. CLS in *D. russelii* bites is associated with myalgia, thirst, parotid swelling, conjunctival chemosis and hypotension^10^. Despite its uniqueness and clinical impact, the phenomenon of CLS induced by *D. russelii* venom has not been explored at the experimental level, and the toxins responsible for this manifestation have not been identified. The experimental study of this phenomenon will contribute to a better understanding of its pathogenesis and pave the way for the development and testing of novel therapeutic approaches for *D. russelii* envenoming. In addition, this knowledge may provide clues for understanding systemic CLS from a wider perspective, since this syndrome occurs in a variety of diseases^11^.

In this study, we describe an experimental model of envenoming by Pakistani *D. russelii* in mice that reproduces the hemoconcentration characteristic of this syndrome, and compare it with the action of the venom of *Bothrops asper*, a pit viper from Central America that induces the archetypical pattern of viperid venom-mediated vasculotoxicity characterized by local and systemic hemorrhage, but does not induce the CLS syndrome^12^. Our observations indicate that this dichotomous pattern of vascular toxicity in viperid venoms is likely to depend on the relative proportion of the action of hemorrhagic SVMPs and of factors causing CLS associated with hemoconcentration.

## Materials and Methods

### Venoms

*Bothrops asper* venom was obtained from more than 40 adult specimens collected in the Pacific region of Costa Rica and kept at the serpentarium of the Instituto Clodomiro Picado. After collection, venom was pooled, lyophilized, and stored at −20 °C until used. *Daboia russelii* venom originating from specimens collected in Pakistan was purchased from LATOXAN (Code L1132A; Lot: 015.051; France). All procedures involving the collection, maintenance and venom extraction from both species of snakes meet the International Guiding Principles for Biomedical Research Involving Animals (CIOMS). Protocols used in Costa Rica for snake handling and venom collection were approved by the Ministry of Environment (resolution number ACCVC-028-OSJ-VS-2015).

### Changes in hematocrit after intramuscular injection of venoms

Groups of five mice (18–20 g) received an intramuscular (i.m.) injection, in the right gastrocnemius, of doses of 10, 15 or 20 µg of *D. russelii* venom, dissolved in 100 µL of 0.12 M NaCl, 0.04 M phosphate, pH 7.2 (PBS) solution. Similarly, five animals were injected with 20 µg/100 µL of *B. asper* venom. Control mice received 100 µL PBS under otherwise identical conditions. One hour after injection, mice were bled, under anesthesia, from the ocular plexus. Blood was collected into heparinized capillary tubes and centrifuged in order to determine the hematocrit, i.e. the percentage of packed red blood cell volume. In addition, the concentration of albumin in plasma was determined by bromocresol green (BCG) colorimetric assay (Alb2: CAN 413) using a Cobas c Analyzer following the instructions of the manufacturer (Roche/Hitachi system). To explore the dynamic nature of envenoming, the time course of alterations induced by *D. russelii* venom in hematocrit was studied. For this, 20 µg venom/100 µL were injected i.m. to groups of 5 mice, as described, and the hematocrit was determined at various time intervals (1 h, 4 h and 24 h). Additionally, as a control, *D. russelii* venom was diluted in normal mouse plasma instead of PBS, and the effect on hematocrit was assessed.

Experiments involving the use of mice were approved by the Institutional Committee for the Care and Use of Laboratory Animals (CICUA) of the University of Costa Rica (permission CICUA-025-15). Experiments involving animals meet the International Guiding Principles for Biomedical Research Involving Animals (CIOMS).

### Venom-induced increment in local vascular permeability

Groups of five mice (18–20 g) received an intravenous (i.v.) injection of 200 μL of a 6 mg/mL Evans blue (EB) solution (60 mg/kg; Sigma-Aldrich). Twenty minutes after EB injection, various doses (2.5, 5 or 10 µg in 50 µL PBS) of either *D. russelii* or *B. asper* venom were injected intradermally (i.d.) in the ventral abdominal region. A control group of mice received 50 µL of PBS. One hour after venom injection, animals were sacrificed by CO_2_ inhalation, their skin was removed, and the areas of EB extravasation in the inner side of the skin were measured.

### Histological assessment of local tissue damage

A dose of 20 µg of either *D. russelii* or *B. asper* venom, dissolved in 50 µL PBS, was injected i.m. in the right gastrocnemius of groups of four mice (18–20 g). Controls received 50 µL of PBS. Three hours after injection, mice were sacrificed by CO_2_ inhalation and a sample of injected gastrocnemius was excised and routinely processed for embedding in paraffin. Sections of 8 µm were obtained and stained with hematoxylin-eosin for histological assessment of tissue damage.

### Inhibition of SVMPs and phospholipases A_2_ (PLA_2_)

In order to evaluate whether SVMPs or PLA_2_s in *D. russelii* venom are responsible for hemoconcentration, groups of five mice (18–20 g) received an i.m. injection of *D. russelii* venom (20 µg/100 µL) in which either SVMPs or PLA_2_s had been inhibited. Inhibition of SVMPs was performed by incubation with the peptidomimetic hydroxamate Batimastat (100 µM, Sigma-Aldrich, MO), as described by Rucavado *et al*.^13^. PLA_2_ activity was inhibited by incubation of venom with *p*-bromophenacyl bromide (pBPB)^14^. Briefly, 3 mg of *D. russelii* venom were dissolved in 1 mL of 0.1 M Tris-HCl containing 0.7 mM EDTA (pH 8.0), and 150 µL of pBPB (1.5 mg/mL, in ethanol) were added. The mixture was then incubated for 24 h at room temperature. Inhibited venom was tested for its ability to induce hemoconcentration, as described above.

### Fractionation of *D. russelii* venom

In order to identify the component(s) responsible for hemoconcentration, *D. russelii* venom was fractionated by molecular exclusion on Sephacryl S-200. Venom (50 mg) diluted in 3 ml of 0.01 M phosphate buffer, pH 7.2, was applied to a column (110 × 2 cm) equilibrated and eluted with 0.01 M phosphate buffer, pH 7.2. Fractions of 5 mL/tube were collected at a flow of 0.25 mL/min. Fractions were diluted and aliquots of 100 µL, containing 20 µg protein, were injected in mice and the hematocrit was determined after 1 h, as described. Fractions showing hemoconcentration activity were analyzed by SDS-PAGE under reducing conditions, using a 12% polyacrylamide gel^15^. After staining with Coomassie Brilliant Blue, protein bands were excised from the gel and submitted to mass spectrometry analysis, as described elsewhere^16^.

The active peak from Sephacryl S-200 column was then fractionated by RP-HPLC using an acetonitrile gradient, as previously described^17^. Fractions were collected, dried in a speed vacuum, dissolved in PBS and tested for hemoconcentration activity. Since no activity was detected in any of the HPLC fractions, the active Sephacryl peak was then separated by ion-exchange chromatography using DEAE-Sepharose column or CM-Sepharose, using a NaCl gradient of 0 to 0.3 M. Again, the peaks obtained from these chromatographies were inactive in terms of hemoconcentrating activity. Since the active Sephacryl peak had a C-type lectin-like (snaclec) component, the Sephacryl active fraction was fractionated in a immunoaffinity chromatography column, using a Sepharose column coupled with rabbit antibodies against aspercetin, a C-type lectin-like protein isolated from the venom of *B. asper*^18^. The fractions showing hemoconcentration activity were submitted to mass spectrometry analysis by LC-MS/MS using a Thermo Electron Orbitrap Velos ETD mass spectrometer, as described elsewhere^16^. The data were analyzed by database searching using the Sequest search algorithm in Proteome Discoverer 1.4.1. The results were exported to Scaffold (version 4.3.2. Proteome Software Inc., Portland, OR, USA) to validate MS/MS based peptide and protein identifications, and to visualize multiple datasets in a comprehensive manner. Proteins were identified in Scaffold with a confidence of 95% or higher.

### Studies with exudates

#### Collection of exudates

Groups of five mice received an i.m. injection, in the right gastrocnemius, of 20 μg or either *D. russelli* or *B. asper* venom, dissolved in 100 μL of PBS. One hour after injection, animals were sacrificed by CO_2_ inhalation, and an incision was made in the skin overlying the injected muscle, as previously described^19^, with care taken to avoid contamination. An heparinized tube was introduced beneath the skin, and the exudate was collected by capillarity. Wound exudates collected were pooled and centrifuged to eliminate erythrocytes and other cells. In some experiments, exudates were lyophilized and stored at −70 °C until used. Exudates were reconstituted to the original volume with water for further analyses.

#### Ability of exudates from envenomated mice to induce hemoconcentration

To study the effect of wound exudate on the hematocrit of mice, exudate samples were diluted 1:2 with PBS and aliquots of 100 µL were injected in groups of five mice either by the i.m. or the intravenous (i.v.) route. Hematocrit was assessed after 1 and 4 h, as described. In the case of *D. russelii* venom, in order to assess whether the effect on hematocrit was due to the presence of venom components in exudates, these were incubated with antivenom [polyspecific antivenom prepared at Instituto Clodomiro Picado against the venoms of *D. russelii*, *Echis carinatus*, *Naja naja* and *Hypnale hypnale* from Sri Lanka, see^20^] at a ratio of 12 mg antivenom per 100 µL exudate. After 30 min incubation at 37 °C, aliquots of 100 µL were injected i.m. in mice and the hematocrit quantified 1 h after injection, as described. Controls of antivenom alone and PBS alone were included.

#### Detection of venom in exudate by Western Blotting

Exudates were collected from mice 1 h after i.m. injection of 20 µg *D. russelii* venom, as described. In parallel, 0.5 µg *D. russelii* venom was added to normal mouse plasma, and dilutions were prepared with water. Twenty microliters of samples of exudate or venom-containing plasma were then separated by SDS-PAGE on 12% acrylamide gels under reducing conditions. After transfer to nitrocellulose membranes, immunodetection *D. russelii* was performed by incubating the membranes overnight at 4 °C with the horse polyclonal antivenom (1:300 dilution). Then, an anti-horse IgG peroxidase conjugate (1:1000; Sigma-Aldrich) was added, followed by the chemiluminescent substrate Lumi-Light (Roche). Images were captured with the ChemiDoc XRS+ System (BioRad) and the analysis was performed with the ImageLab software (BioRad).

#### Quantitative determination of inflammatory mediators in exudates induced by *D. russelli* and *B. asper* venoms

The concentrations of a set of cytokines and chemokines were determined in exudates collected from mice 1 h after i.m. injection of 20 µg of either *D. russelii* or *B. asper* venom. Exudate samples collected from five mice injected with each venom were pooled. Quantification was performed by Luminex Assays (Mouse Premixed Multi-Analyte Kit, R&D systems, Minneapolis, MN) following the methodology recommended by the manufacturer. Since no exudate develops from control mice receiving PBS injections, plasma from normal mice was used as control.

#### Statistical analyses

The significance of the differences of mean values between experimental groups was assessed by analysis of variance (ANOVA), followed by Tukey-Kramer test to compare pairs of means. A p value <0.05 was considered significant. In some of the assays Student’s *t* test was used to compare pairs of groups.

## Results

### *D. russelii* venom induces hemoconcentration and hypoalbuminemia

**Figure 1:**
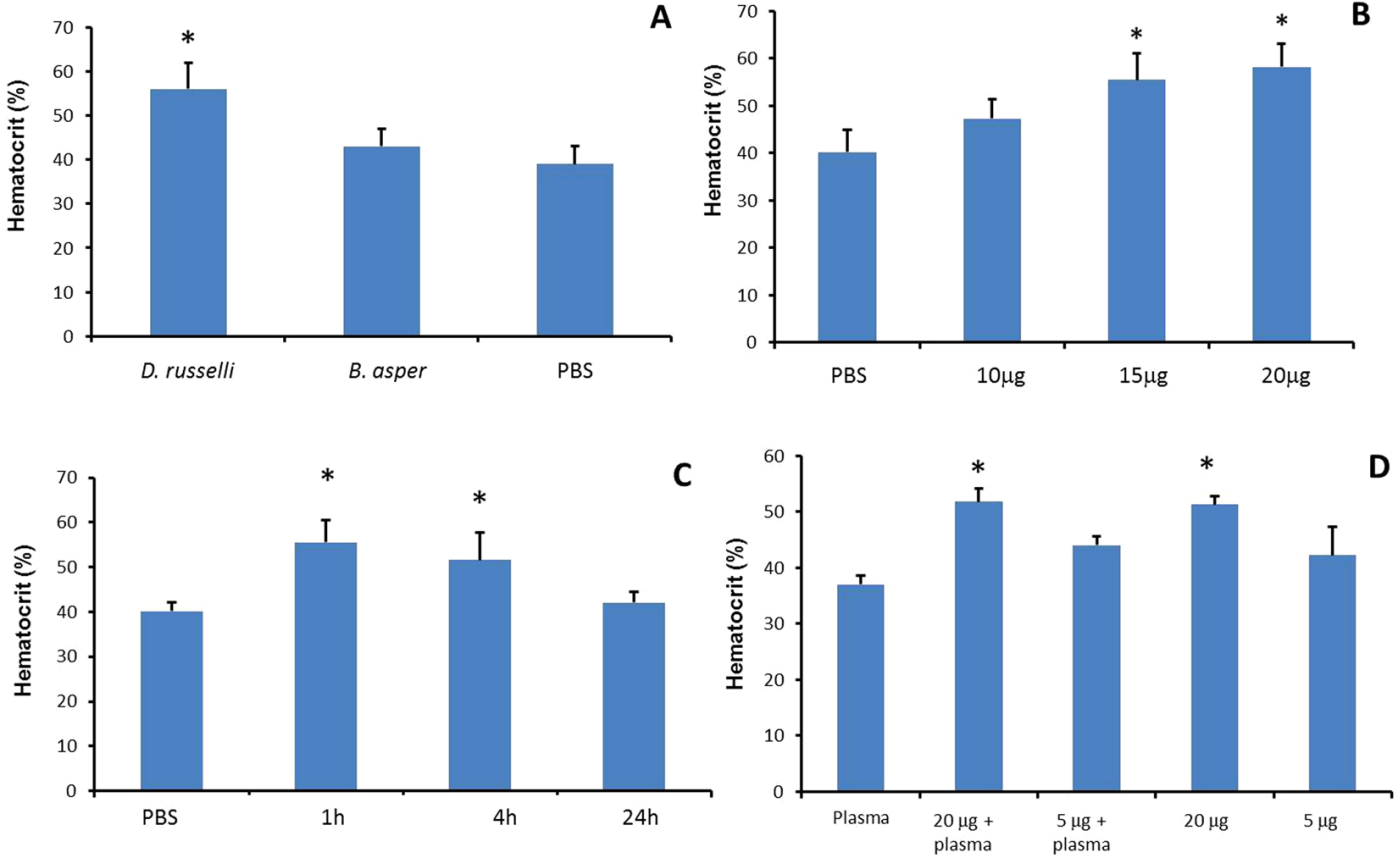
Hemoconcentration induced by *D. russelii* venom. (**A**) Mice received an i.m. injection, in the right gastrocnemius, of 20 µg of *D. russelii* or *B. asper* venom, dissolved in 100 µL PBS, and hematocrit was determined 1 h after injection. Controls received PBS alone. Only *D. russelii* venom induced hemoconcentration. (**B**) When mice received various doses of *D. russelii* venom, under otherwise identical conditions, hemoconcentration developed with the dose of 15 µg and higher. (**C**) Time course of hemoconcentration after i.m. injection of 20 µg of *D. russelii* venom in mice. (**D**) Effect of plasma on the hemoconcentration effect. When injected i.m., plasma itself did not induce the effect. The increases in hematocrit induced by 20 µg *D. russelii* venom diluted either in PBS or plasma were essentially equivalent. Results are presented as mean ± S.D. (n = 5). In A, B and C, *p < 0.05 when compared to hematocrit of mice receiving PBS alone. In D, *p < 0.05 when compared to the other treatments.

One hour after the i.m. injection of 20 µg venom, mice receiving *D. russelii* venom developed hemoconcentration, as indicated by a significant increase in hematocrit. Hematocrit was increased in all animals injected with venom. In contrast, the hematocrit of mice receiving the same amount of *B. asper* venom did not differ from that of mice injected with PBS (Fig. 1A). This effect was dose-dependent, 15 µg of *D. russelii* venom being the minimum dose inducing a significant increment in hematocrit (Fig. 1B). This effect was observed at 1 and 4 h. At 24 h the value of hematocrit had returned to normal levels (Fig. 1C). A similar effect was observed when *D. russelii* venom was dissolved in PBS or in normal mouse plasma (Fig. 1D). Both venoms induced a decrease in albumin concentration in plasma, but the reduction was significantly higher in samples from mice injected with *D. russelii* venom (PBS: 27.3 ± 1.3 g/L; *B. asper*: 20.1 ± 1.9 g/L; *D. russelii*: 17.7 ± 1.2 g/L; p < 0.05 when comparing PBS vs venoms and when comparing the two venoms).

### *D. russelii* and *B. asper* venoms differ in their ability to induce hemorrhage and affect vascular permeability

**Figure 2:**
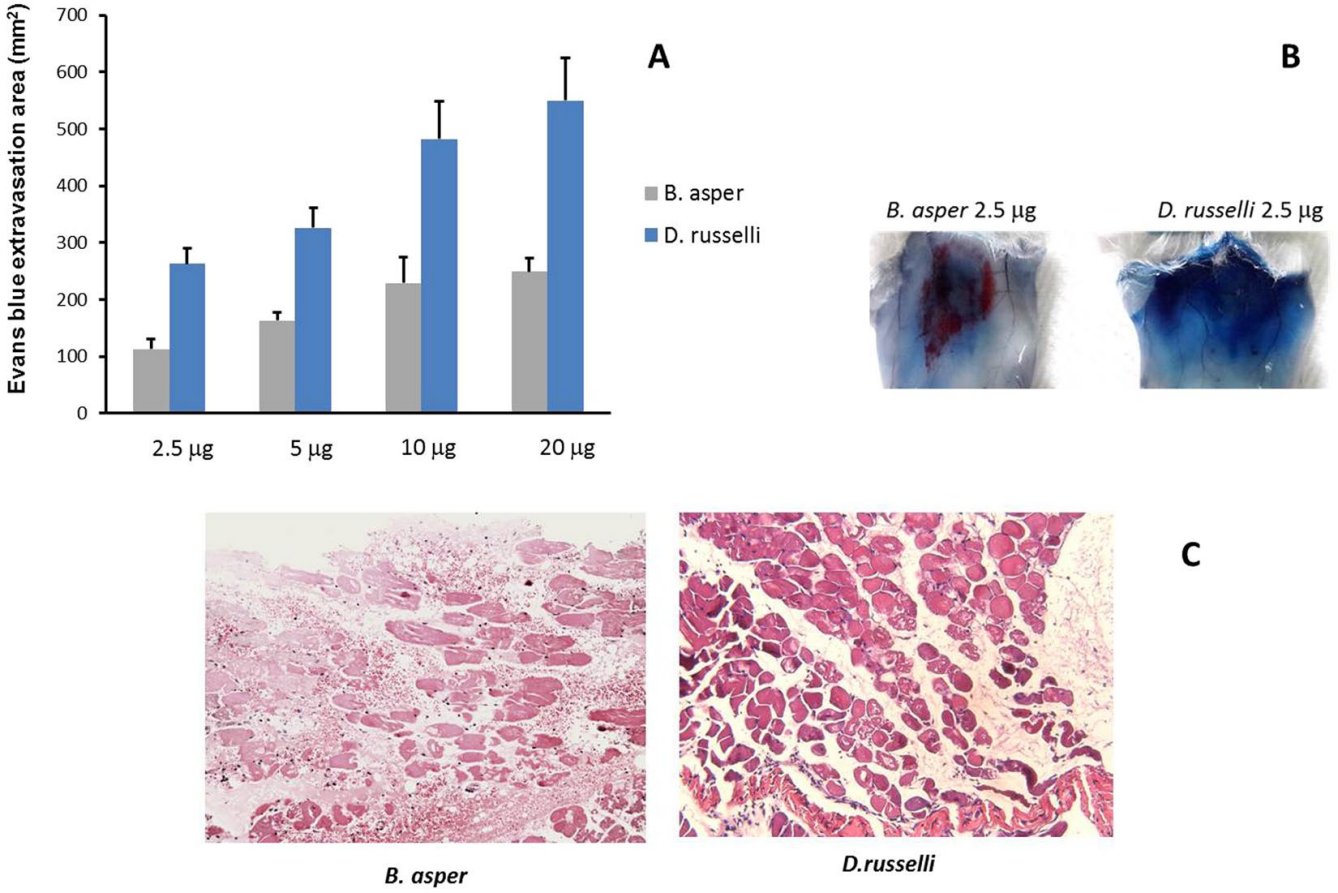
Effect of venoms on vascular permeability. (**A**) and (**B**) Increments in vascular permeability in the skin of mice 1 h after intradermal injection of various doses of *D. russelii* or *B. asper* venoms. Increase in vascular permeability was assessed by extravasation of Evans Blue (see Materials and Methods for details). A higher increment in permeability was observed for *D. russelii* venom at all doses tested; results are presented as mean ± S.D. (n = 5); *p < 0.05. (**B**) Shows the macroscopic appearance of the inner side of the skin 1 h after venom injection of 2.5 µg. Notice the prominent hemorrhagic lesion in the case of *B. asper* venom (red coloration), with little Evans Blue extravasation, whereas a large area of Evans Blue extravasation is observed in the case of *D. russelii*, with no hemorrhage. (**C**) Light micrographs of sections of muscle tissue from mice; tissue was collected 3 h after injection of 20 µg of *B. asper* or *D. russelii* venoms. Widespread hemorrhage is observed in the tissue of *B. asper* venom-injected mice (arrows), whereas no hemorrhage occurred in mice receiving *D. russelii* venom. Hematoxylin-eosin staining. Bar corresponds to 100 µm.

The venom of *D. russelii* induced a higher increment in vascular permeability in the skin than the venom of *B. asper* (Fig. 2A). At a dose of 2.5 µg venom, *B. asper* induced an area of hemorrhage, whereas *D. russelii* did not induce hemorrhage but did cause an increment in the extravasation of Evans Blue (Fig. 2B). Moreover, the venom of *D. russelii*, even at doses up to 20 µg, did not demonstrate hemorrhagic activity. This is in agreement with the histological analysis of gastrocnemius muscle injected with 20 µg venoms, where hemorrhage was observed in muscle injected with *B. asper* venom (Fig. 2C) but not with *D. russelii* venom (Fig. 2C). Thus, these venoms greatly differ in their vasculotoxic effects, *D. russelii* having a higher ability to increase vascular permeability and plasma extravasation, whereas *B. asper* exerts a stronger hemorrhagic activity.

### Identification of venom components responsible for the hemoconcentration effect

**Figure 3:**
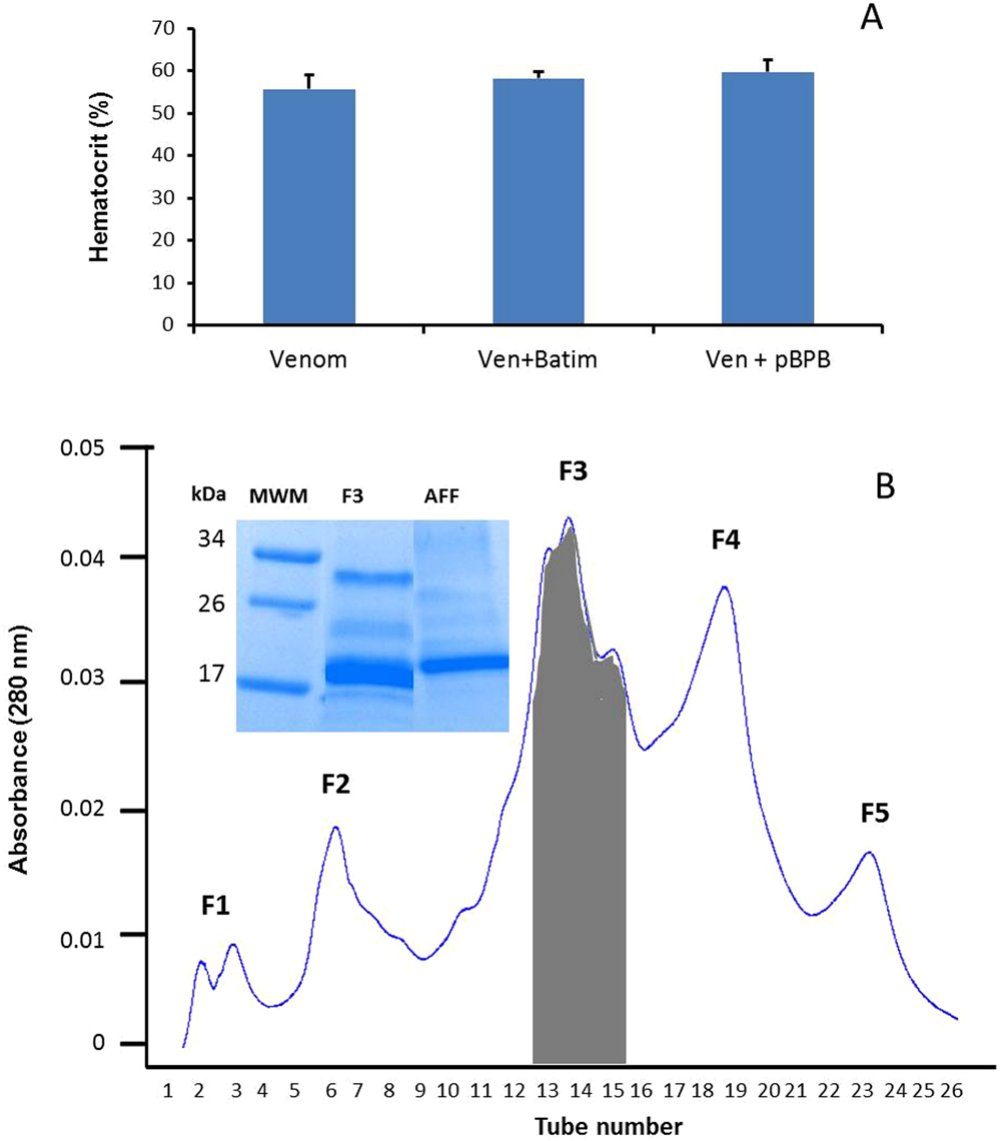
Inhibition of enzymatic activities and fractionation of *D. russelii* venom. (**A**) Effect of inhibition of SVMPs and PLA_2_s of *D. russelii* venom on the hemoconcentration effect. Venom was incubated with Batimastat or p-BPB for inhibition of SVMPs and PLA_2_s, respectively (see Materials and Methods for details). Venom control corresponds to venom incubated with the vehicle solutions used in the inhibition experiments. Samples of native or inhibited venom (20 µg) were injected i.m. into mice and hematocrit was quantified 1 h after injection. No reduction of the effect was achieved with these inhibitors. (**B**) Fractionation of *D. russelii* venom by gel filtration. Venom (50 mg) was dissolved in 3 mL of phosphate buffer, pH 7.2, and applied to a Sephacryl S-200 column (110 × 2 cm). Only fraction 3 induced hemoconcentration. Insert in 3B shows SDS-PAGE (reducing conditions) of peak 3 of Sephacryl (F3) and of the peak obtained from the affinity chromatography (AFF). Three bands of molecular masses of 16, 26 and 32 kDa were detected in fraction 3 of Sephacryl, whereas one predominant band of about 17 kDa waspresent in the affinity-purified peak. MWM: Molecular weight markers.

When *D. russelii* venom was incubated with inhibitors of SVMPs or PLA_2_s, the hemoconcentration effect was not reduced (Fig. 3A), implying that enzymatic activity of SVMPs or PLA_2_s does not directly play a key role in this phenomenon. Separation of *D. russelii* venom on Sephacryl S-200 yielded four peaks (Fig. 3B) of which only peak 3 caused hemoconcentration. Upon separation of the proteins of this peak by SDS-PAGE under reducing conditions, three bands of 16, 26 and 32 kDa were identified (Insert Fig. 3B).

Mass spectrometry analysis of these bands revealed internal sequences having identity with the following proteins in the databank: 15 kDa band, basic PLA_2_, acidic PLA_2_, Kunitz-type proteinase inhibitor, vascular endothelial growth factor (VEGF), C-type lectin-like protein; 26 kDa band, Factor V activator (RVV-V), basic PLA_2_, acidic PLA2, P-I SVMP, cysteine-rich secretory protein (CRISP); and 32 kDa band, RVV-V, P-I SVMP, and serine proteinase. Proteins present in peak 3 were then separated by RP-HPLC, and 12 peaks were obtained. None of these peaks induced hemoconcentration when tested at a dose of 20 µg. Surprisingly, when Sephacryl peak 3 was fractionated by ion-exchange chromatography on CM-Sepharose and DEAE-Sepharose, no hemoconcentrating activity was detected in any of the peaks. Since mass spectrometry analysis identified a C-type lectin-like component in peak 3, this fraction was submitted to an affinity-chromatography column using rabbit antibodies raised against aspercetin, a snaclec from *B. asper* venom, which cross-reacted by dot blot against peak 3. A protein fraction, having a predominant band of 17 kDa (Fig. 3B), bound to the column and induced hemoconcentration in mice. This fraction contains, on the basis of mass spectrometry analysis, a C-type lectin-like (snaclec) (SLA_DABSI), venom VEGF (TXVE_DABRR) and an acidic PLA_2_ (PA2A7_VIPAA) (Table 1). The relative amounts of each protein in this fraction, as judged by mass spectrometry, were 3: 2.6: 1 for snaclec: acidic PLA_2_: VEGF.

**Table 1:** Proteins identified by mass spectrometry in the fraction obtained from affinity chromatography using anti-snaclec antibody.

Protein	Accession	Mass (kDa)	m/z	Z	MS-MS peptide sequence	Sequest XCorr score	Coverage %
Snaclec dabocetin subunit alpha	SLA_DABSI (Q38L02)	17.506					34
			439.70	2	TWEDAEK	2.23	
			508.28	2	YHAWIGLR	2.47	
			757.67	3	QQCSSHWTDGSAVSYETVTK	5.66	
			570.28	4	YHEWITLPCGDKNPFICK	3.24	
Snake venom vascular endothelial growth factor toxin VR-1	TXVE_DABRR (P67861)	16.277					30
			667.73	2	CSGCCTDESMK	2.48	
			542.70	2	HTADIQIMR	2.89	
			531.9	2	FMEHTACECRPR	2.59	
			408.54	2	QGEPEGPKEPR	2.79	
Acidic phospholipase A2 RV-7	PA2A7_DABSI (P31100)	15.429					36
			769.29	3	CCFVHDCCYGTVNDCNPK	5.40	
			819.40	2	AAAICLGQNVNTYDK	3.68	
			1099.92	2	NYEYYSISHCTEESEQC	3.53	

### Exudates collected from envenomated mice induce hemoconcentration

**Figure 4:**
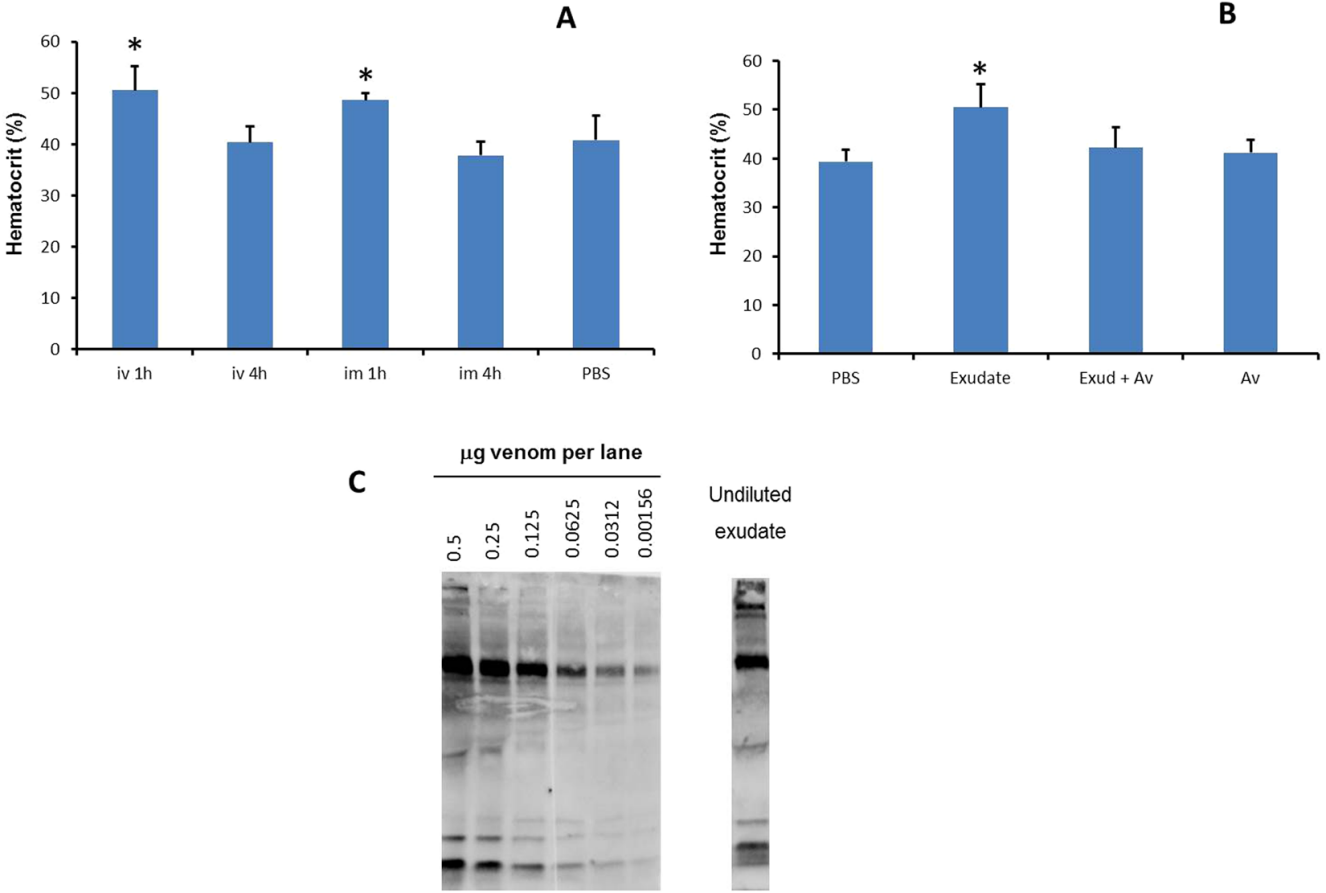
Effect on hematocrit of exudates collected from envenomed mice. (**A**) A dose of 20 µg of *D. russelii* venom, dissolved in 100 µL PBS, was injected i.m. in mice. One hour after injection, groups of mice were sacrificed and a sample of inflammatory exudate was collected. Exudates from each experimental group were pooled. After centrifugation to eliminate cells, the exudates were diluted 1:2 with PBS and aliquots of 100 µL were injected wither i.m. or i.v. into mice. Hematocrit was quantified 1 and 4 h after exudate injection. Control mice received 100 µL of PBS by the i.m. route. (**B**) Inhibition of the hemoconcentration effect of exudate by incubation with antivenom. Exudates collected as described were incubated with either PBS or polyspecific antivenom at a ratio of 12 mg antivenom per 100 µL exudate. After 30 min incubation at 37 °C, aliquots of 100 µL of the mixtures were injected i.m. in mice, and hematocrit was quantified 1 h after injection. Controls included mice receiving PBS alone, antivenom alone, or exudate incubated with PBS instead of antivenom. Results are presented as mean ± S.D. (n = 5). *p < 0.05 as compared to PBS-treated mice. (**C**) Immunoblotting of samples of various amounts of *D. russelii* venom, dissolved in normal mouse plasma, and of an aliquot of 20 µL of exudate collected from mice 1 h after injection of 20 µg *D. russelii* venom. Samples were separated by SDS-PAGE in 12% acrylamide gels under reducing conditions and, after transfer, the nitrocellulose strip was incubated with polyspecific antivenom and revealed by anti-horse IgG peroxidase conjugate and a chemiluminiscent substrate (see Materials and Methods for details). All lanes of the left figure correspond to an uncropped gel; the right lane (exudate) corresponds to a different gel ran at the same time as the left side gel and revealed under identical conditions and exposure time.

Exudates were collected from mice 1 h after injection of 20 µg *D. russelii* or *B. asper* venoms. Exudates from mice injected with *B. asper* venom had a reddish appearance, whereas those from *D. russelii*-injected mice were slightly yellowish. When these exudates were injected into mice by either the i.m. or the i.v. routes, and hematocrit quantified 1 and 4 h later, there was a significant hemoconcentration at 1 h in the case of exudates from mice injected with *D. russelii* venom (Fig. 4A), but not in those from animals receiving *B. asper* venom. In order to assess whether this effect was due to the action of venom components present in the exudate, the latter was incubated with antivenom and then tested; under these conditions the hemoconcentration effect was inhibited (Fig. 4B), thus indicating that venom proteins in the exudate were largely responsible for the effect. The presence of venom proteins in the exudate was demonstrated by immunoblotting (Fig. 4C). A semiquantitative estimation of the amount of venom present in the exudate was performed (Fig. 4C). Results show that between 0.5 µg and 0.25 µg venom were present in the sample of 20 µL exudate ran in the SDS-PAGE gel. Thus, since 100 µL of a 1:2 dilution of exudate was injected in mice, corresponding to an absolute volume of 50 µL exudate, the estimated amount of venom injected ranged between 1.25 µg and 0.62 µg (Fig. 4C), whereas the minimal dose of venom diluted in PBS required to induce the hemoconcentration effect was 15 µg. In addition, the main immunoreactive bands detected in the venom were also present in the exudate sample. Thus, these observations suggest that some components of the exudate have a potentiating effect for inducing hemoconcentration, although the effect is blocked when venom is inhibited. Hence, as a follow up, the analysis of the presence of inflammatory mediators in the exudate was performed.

### Analysis of cytokines and chemokines in exudates

The analysis of the subproteome of cytokines and chemokines in exudates revealed a complex pattern. For some mediators, there was not an increment in concentration in exudates as compared to normal plasma, suggesting that their presence in exudate is likely to be a consequence of plasma extravasation. In contrast, other mediators showed a higher concentration in exudates than in normal plasma. Among these, exudates collected from mice injected with *B. asper* venom showed in general higher amounts of cytokines and chemokines, especially in the case of IL-6, IL-10, KC, MCP-1 and MIP-2, than exudates from mice injected with *D. russelii* venom. The latter, in turn, had higher concentrations of IL-17 and IL-9 (Table 2). Mouse VEGF was detected in exudates collected from mice injected with the two venoms, with a slightly higher amount in the case of *D. russelii* (Table 2).

**Table 2:** Concentrations of cytokines and chemokines in normal mouse plasma and in pools of exudates collected from mice 1 h after i.m. injection of 20 µg of either *D. russelii* or *B. asper* venoms. Quantification was performed by Luminex Assays.

Analyte pg/ml	Plasma	DR exudate	BA exudate
G-CSF	1231	878.12	1840
EOTAXIN	441.81	136.53	269.03
GM-CSF	23.91	7.96	14.36
IFNy	0.96	3.44	<0.15
IL-1a	183.44	322.97	482.73
IL-1b	4.81	9.46	17.96
IL-2	1.17	4.77	4.16
IL-4	<0.14	<0.14	0.24
IL-3	0.45	0.21	0.59
IL-5	9.99	9.57	9.75
IL-6	6.47	8881	>16347
IL-7	<0.72	104.38	1.05
IL-9	40.28	419.34	100.06
IL-10	10.52	340.08	1875
IL-12p40	2.75	12.61	11.24
IL-12p70	2.49	4.17	5.37
LIF	3.61	12.76	18.79
IL-13	33.07	66.2	70.37
LIX	2687	604.22	578.41
IL-15	<3.20	20.24	53.83
IL-17	2.18	0.86	1.16
IP-10	274.58	58.01	154.05
KC	247.16	2603	6722
MCP-1	5.69	383.39	738.55
MIP-1a	31.14	45.18	103.88
MIP-1b	34.22	75.39	533.59
M-CSF	6.23	11.58	15.14
MIP-2	68.62	2611	>12977
MIG	139.8	127.02	132.05
RANTES	21.7	8.18	10.16
VEGF	0.26	1.22	0.72
TNF-a	11.14	10.11	46.16

## Discussion

To follow up on previous investigations on the ability of *D. russelli* to induce increments in local vascular permeability, we developed a simple experimental model to study the CLS induced by the venom of *D. russelii* from Pakistan, based on the determination of blood hematocrit and plasma albumin concentration in mice. Since CLS is associated with a massive increment of vascular permeability leading to plasma extravasation and hypoalbuminemia, one consequence of this effect is hemoconcentration, as described in clinical cases of envenomings by *D. russelii*^8,9^. In contrast, no hemoconcentration was observed in mice upon injection of the venom of *B. asper*. CLS has, at least, two serious pathophysiological consequences in envenomings: it contributes to hypovolemia leading to cardiovascular collapse and shock, and hemoconcentration causes an increase in blood viscosity^21^, reducing blood flow and thus impairing perfusion to organs and contributing to organ failure. Accordingly, hemoconcentration has been associated with severity in envenomings by some populations of *D. russelii*^8–10^, although there are notorious variations in the clinical manifestations of envenomings by diverse populations of this species^7^. Thus, the relevance of the CLS in the overall clinical context of envenomings by *D. russelii* varies depending on the region and no generalizations can be made.

At a relatively low dose, the venoms of *D. russelii* from Pakistan and of *B. asper* induced two different patterns of vascular toxicity in our model. When injected intradermally, *B. asper* venom caused a local hemorrhagic lesion, whereas *D. russelii* did not elicit hemorrhage but instead induced an increment in vascular permeability, as revealed by Evans Blue extravasation. Likewise, histological examination of muscle tissue injected with these venoms confirmed this difference, since *B. asper* venom induced hemorrhage while *D. russelii* venom from Pakistan was devoid of this effect at the dose tested. This reveals two distinct paradigms of vascular toxicity, one based on disruption of microvascular network leading to hemorrhage (*B. asper*) and the other centered in an increment in vascular permeability leading to hemoconcentration (*D. russelii* from Pakistan). This difference may also have implications in the mechanisms of systemic absorption of venom components from the locale of venom injection. It is likely that the lymphatic route of absorption is followed by both venoms, although the vascular route is likely to be impaired in the case of *B. asper* venom owing to the disruption in the capillary network, i.e. hemorrhage.

We suggest that this dichotomous pattern of vascular alterations described depends on the composition of the venoms. Despite recognized intraspecies variations in the composition of venoms of Russell’s viper from different geographical origins, a common proteomic trend is the relative low percentage of SVMPs, although there is a notorious intraspecies variability^22–28^. Moreover, no hemorrhagic activity was observed in the skin and muscle of mice after injection of 20 µg of *D. russelii* venom from Pakistan^29^ and this work. In contrast, the venom of *B. asper* has a high content of SVMPs^30^, some of which are hemorrhagic^31,32^, and induced evident hemorrhagic lesions in the skin and muscle of mice. In agreement, inhibition of SVMPs by Batimastat did not abrogate *D. russelii*-induced hemoconcentration, hence indicating that this effect is not directly due to SVMPs.

Nevertheless, owing to the demonstrated intraspecies variability in the composition of *D. russelii* venoms, there are populations of this species in which SVMPs-induced hemorrhagic activity occurs, as shown in the clinical setting^7,33^. Thus, the vascular effects induced by the venom of *D. russelii* from Pakistan used in this study cannot be generalized as being the predominant vasculotoxic activity of venoms of this species from other populations, since *D. russelii* venoms from specimens collected in other regions of Asia, as well as the venom of the closely related species *D. siamensis*, have been described as being hemorrhagic and containing higher amounts of SVMPs^20,26–28^. It would be relevant to study Russell’s viper venoms from diverse geographical origins in order to determine which ones induce the CLS described in this work, and to correlate these observations with the predominant clinical features of envenomings.

Both venoms used in this study have a high content of PLA_2_^22–25,30^. PLA_2_s play a significant role in the overall toxicity of *D. russelii* venom, since they induce neurotoxicity, myotoxicity, edematogenic activity, and other effects^34–36^. However, inhibition of PLA_2_ activity by *p*BPB did not affect the hemoconcentration effect, thus suggesting that the responsible component for the CLS is unlikely to be a catalytically-active PLA_2_.

Fractionation of *D. russelii* venom, with the aim of identifying the toxin(s) responsible for hemoconcentration, was initially performed by gel filtration on Sephacryl S-200. The active peak was then separated by RP-HPLC and anion- and cation-exchange chromatography but, unexpectedly, the hemoconcentration activity was not found in any peak. We then used an affinity chromatography with an anti-snaclec antibody, and the fraction that bound to the antibodies induced hemoconcentration. When analyzed by mass spectrometry, this peak contained, in addition to a snaclec, venom VEGF and a PLA_2_. Proteomic analyses of *D. russelii* venom from various geographical regions, including Pakistan, have shown the presence of VEGF^22,23,25,29^, in contrast with the venom of *B. asper* in which this component has not been reported^30^. Previous studies have demonstrated that VEGF from the venoms of various viperid species, including *D. russelii*, exerts a potent hypotensive effect and induces increments in vascular permeability in the skin at very low doses^37–39^. VEGF from snake venom binds to KDR receptor in endothelial cells^37^ and induces increments in vascular permeability predominantly by the formation of fenestrae in these cells^40^. We therefore suggest that VEGF, which is present in the proteome of *D. russelii* venom from Pakistan^25,29^ is the main responsible for the hemoconcentration effect and CLS induced by *D. russelii* in this experimental model, as a consequence of the increment that it induces in vascular permeability. Further studies with purified components are necessary to confirm this hypothesis.

Exudates collected from the site of *D. russelii* venom injection also elicited hemoconcentration upon i.m. and i.v. injections. Most venom components were present in these exudates, as judged by Western blot, and neutralization of venom by antivenom completely abrogated the hemoconcreation effect of the exudate, indicating that venom proteins in the exudate are responsible for the activity. However, the estimated amount of venom in the injected volume of exudate was much lower than the minimum amount of venom necessary to elicit hemoconcentration when dissolved in PBS. This suggests that components of the exudate may potentiate the action of venom. Furthermore, exudate contains high amounts of several chemokines and cytokines, and low amounts of mouse VEGF, all of which could act on endothelial cells, thus potentiating the effect of venom VEGF. Thus, the potent vascular activity of venom VEGF is likely to be potentiated by components in exudate such as cytokines and chemokines, and studies are underway in our laboratory to further elucidate this possibility.

In conclusion, a mouse model has been developed to investigate the CLS induced by *D. russelii* venom from Pakistan, a significant feature in the complex pathophysiology of envenomings by this snake. Our results suggest that the increment in vascular permeability induced by this venom is likely to be potentiated by endogenous components present in the exudate generated as a consequence of envenoming. In the case of *B. asper* venom, the high concentration of hemorrhagic SVMPs is responsible for a predominant vasculotoxicity associated with microvascular damage leading to hemorrhage, and not with a systemic increase in vascular permeability and hemoconcentration. Owing to the high regional variation in the composition of *D. russelii* venoms, it would be relevant to assess whether venoms from other populations are also able to induce CLS and hemoconcentration, to further define this dichotomy of viper venom-induced pathophysiology.

## Data Availability

The datasets generated during and/or analysed during the current study are available from the corresponding author on reasonable request.
